# Pancreatic involvement in *EPG5*-related disorders

**DOI:** 10.1016/j.ymgmr.2025.101273

**Published:** 2025-11-10

**Authors:** Dennis T. Famili, Gehad Elghazali, Emanuela Argili, Russell P. Saneto, Michael Harris, Oleg Gerasimenko, Julia Gerasimenko, Manolis Fanto, Hormos Salimi Dafsari, Heinz Jungbluth

**Affiliations:** a Department of Paediatric Neurology, Neuromuscular Service, Evelina's Children Hospital, Guy's & St. Thomas' Hospital NHS Foundation Trust, London, United Kingdom; b Sheikh Khalifa Medical City, Abu Dhabi, United Arab Emirates; c Brain Development Research Program, UCSF, San Francisco CA94158, USA; d Seattle Children's Research Hospital, Seattle, WA 98105, USA; e Vici Syndrome Foundation, USA; f School of Biosciences, Cardiff University, Cardiff, United Kingdom; g Department of Basic and Clinical Neuroscience, IoPPN, King's College London, United Kingdom; h Department of Pediatrics and Center for Rare Diseases, Faculty of Medicine and University Hospital Cologne, University of Cologne, Germany; i Max-Planck-Institute for Biology of Ageing, Cologne Excellence Cluster on Cellular Stress Responses in Aging Associated Diseases (CECAD), Cologne, Germany; j Randall Centre for Cell and Molecular Biophysics, Muscle Signalling Section, Faculty of Life Sciences and Medicine (FoLSM), King's College London, London, United Kingdom

**Keywords:** Pancreatitis, EPG5, Autophagy, Intracellular trafficking

## Abstract

Vici syndrome is a severe neurodevelopmental multisystem disorder characterized by callosal agenesis, cataracts, cardiomyopathy, combined immunodeficiency and hypopigmentation. There may be additional variable involvement of other organs. *VS* is caused by recessive mutations in *EPG5*, encoding a tethering factor with important roles in autophagy, an essential cellular homeostatic mechanism involved in metabolic adaptation, infection defence and quality control of proteins and organelles.

Acute pancreatitis is an inflammatory syndrome caused by an acute injury resulting in failure of safeguarding mechanisms preventing autodigestion. Chronic pancreatitis is characterized by replacement of pancreatic parenchyma with fibrotic tissue following repeated injury, resulting in endocrine and exocrine insufficiency. In addition to common causes such as excessive ethanol consumption, gallstones and pharmacological factors, there are likely to be additional genetic contributors.

Here we report 3 patients with *EPG5*-related Vici syndrome and not previously recognized pancreatic involvement, ranging from otherwise asymptomatic amylase elevations to acute pancreatitis and pancreatic insufficiency. A topical literature review on the role of autophagy and autophagy-related genes in the pancreas suggested that autophagy defects may affect critical pathological events involved in pancreatitis, in particular abnormal vacuole formation in acinar cells, inappropriate intra-acinar trypsinogen activation, mitochondrial dysfunction and disturbed calcium homeostasis.

These findings illustrate the importance of *EPG5* and other autophagy-related genes in normal pancreatic function and expand the phenotypical spectrum of *EPG5*-related disorders.

## 1 Introduction

Vici syndrome, initially described by Dionisi Vici in 1988 [1], is a severe neurodevelopmental and neurometabolic disorder characterized by the key features of callosal agenesis, cataracts, combined immunodeficiency, cardiomyopathy and hypopigmentation.

Vici syndrome has been attributed to recessive mutations in *EPG5* [2,3], encoding ectopic P-granules 5 protein with a crucial role in autophagy, an essential cellular homeostatic mechanism involved in metabolic adaptation, infection defence, and quality control of proteins and organelles, in particular mitochondria. The phenotypical spectrum of *EPG5*-related disorders continues to expand and now includes a much wider range of neurodevelopmental [4] and neurological disorders including early onset Parkinsonism [5]. Whilst the focus so far has been on the detailed characterization of *EPG5*-associated neurodevelopmental and neurological features, additional non-neurological features are expected considering the fundamental role of autophagy and the ubiquitous expression of the EPG5 protein in non-neuronal tissues.

Acute pancreatitis is an inflammatory syndrome of the pancreas caused by an acute injury [6], with a broad spectrum of severity [7]. Recurrent episodes develop in 21 % of patients, with 36 % developing chronic pancreatitis [8]. Typical features include persistent and severe abdominal pain radiating into the back, increases in serum amylase and/or lipase level and suggestive abdominal CT findings; at least two of these features are required to establish the diagnosis [9]. Diagnosis of pancreatitis in patients with severe neurodevelopmental disorders requires a high degree of suspicion as those may not be able to vocalize potentially diagnostic complaints. Common causes in the first world include excessive ethanol consumption, gallstones, metabolic abnormalities, and iatrogenic, in particular pharmacological factors [10]. An acute pancreatic insult appears to cause a failure of safeguarding mechanisms preventing autodigestion, leading to the inappropriate intrapancreatic activation of proteases [11], particularly trypsin [12]. These initial events may trigger necrosis of pancreatic parenchymal cells and a subsequent inflammatory response, with the local intra-pancreatic and/or systemic recruitment of inflammatory cells, leading to multi-organ dysfunction [13]. Chronic pancreatitis, on the other hand, is a fibroinflammatory disorder where repeated episodes of pancreatic injury and inflammation result in the extensive replacement of pancreatic parenchyma with fibrotic tissue, with patients often suffering from chronic pain, endocrine insufficiency (in the form of diabetes mellitus) and exocrine insufficiency (in the form of gastrointestinal malabsorption and nutritional deficiencies, potentially resulting in weight loss and growth restriction) [14]. The causes of chronic pancreatitis include, among others, excessive ethanol exposure, metabolic abnormalities, and genetic factors [15]. Pancreatic involvement may also be a feature in rare neurodevelopmental and neurometabolic, for example mitochondrial disorders; investigation of such disorders may elucidate key mechanisms implicated in acute and chronic pancreatitis that are currently not well-understood.

Here we present 3 patients with an *EPG5*-related disorder and not previously reported pancreatic involvement, ranging from otherwise asymptomatic amylase elevations to acute pancreatitis and pancreatic insufficiency. We also review the literature on the role of autophagy in the pancreas, and more specifically in the pathophysiology of acute and chronic pancreatitis. Our original findings and the literature review illustrate the important role of EPG5 and other autophagy-related proteins in normal and abnormal pancreatic physiology. Those findings also expand the phenotypical spectrum of *EPG5*-related disorders, with important implications for the health surveillance of patients affected by these conditions.

## 2 Methods

Patients were recruited through an established international collaboration dedicated to the investigation of Vici syndrome and other *EPG5*-related disorders [detailed in reference [5].

Genetic testing (through whole exome sequencing) had been performed as part of the routine diagnostic process. Patients were managed by a multidisciplinary team as recommended for patients with *EPG5*-related disorders [16], tailored according to their individual needs.

The study was approved by the ethics committee of the Medical Faculty, University of Cologne (20–1711) and other local institutional review boards. All patients and/or their legal guardians gave informed consent to anonymized publication and use of recognizable photographs/videos where applicable.

## 3 Case histories

### 3.1 Case 1

Patient 1 is a 4-year-old male with an established diagnosis of Vici syndrome due to compound heterozygosity for *EPG5* variants NC_000018.10:g.45916107C > A;NM_020964.3:c.3484G > T;NP_066015.2:p.Glu1162Ter and NC_000018.10:g.45944005C > A;NM_020964.3c.1792G > T;NP_066015.2:p.Gly598Cys. He had profound neurological features including global developmental delay, callosal agenesis and microcephaly (with a head circumference more than 2.5 standard deviations below the lower limit of normal). He also had cortical visual impairment. He had generalized hypertonia with multiple contractures but diminished deep tendon reflexes. He suffered from refractory epilepsy for which he was being treated with carbamazepine after exhaustive trials of a range of established anticonvulsants. He also suffered from hearing loss of unknown type. He had a history of cardiorespiratory arrest at year 1 of age. He was dependent on nasogastric tube feeding due to impaired swallow and had chronic acid reflux. He had chronic *C. difficile* colonisation. Non-contrast magnetic resonance imaging (MRI) of the brain, performed at age 4 years and 3 months was consistent with Vici syndrome, demonstrating absence of the corpus callosum, reduced white matter content, areas of anomalous cortical folding and hypoplasia of the cerebellar vermis, pons and brainstem.

On the background of his neurodevelopmental disorder, at the age of 4 years he presented with pyrexia and abdominal pain, following 14 days of antibiotic treatment for a urinary tract infection caused by *Citrobacter.* Investigations on admission revealed markedly elevated serum amylase (508 IU/L, reference range 0–115) and lipase (4053 IU/L, reference range 25–120) levels, consistent with acute pancreatitis. An abdominal ultrasound scan revealed no evidence of ductal dilation or biliary stones. Anterior mesenteric oedema extending posteriorly to the pancreas but without pancreatic pseudocysts was demonstrated on abdominal computer tomography (CT). These findings were confirmed on abdominal MRI which did not reveal any additional abnormalities. The pancreatitis was attributed to carbamazepine treatment which was consequently discontinued. Serum amylase and lipase levels gradually declined (260 and 544 IU/L, respectively, on day 4) but increased again (to 900 IU/L and 1244 IU/L respectively) following re-initiation of oral feeding. He was subsequently changed to total parenteral nutrition followed by only slow improvement of laboratory markers. Repeat laboratory investigations on day 51 revealed a lipase level of 710 IU/L; this remained elevated for a year following the acute episode before returning to normal.

### 3.2 Case 2

The second patient was a three-month-old male who had an established diagnosis of Vici syndrome. He was found to have a complex *EPG5* genotype, with heterozygosity for *EPG5* NC_000018.10:g.45934833C > T;NM_020964.3:c.2233G > A;NP_066015.2:p.Ala745Thr variant and homozygosity for the *EPG5* NC_000018.10:g.45943325 A > T;NM_020964.3:c.1793-14 T > A variant, 14 nucleotides upstream of the splice acceptor site for exon 9 and thought to disrupt the canonical splice acceptor site by creating a new splice acceptor site. He was globally delayed. He was fully fed *via* a gastrojejunostomy tube (which was also used for his medication) and had gastro-oesophageal reflux disease treated with Omeprazole.

On above background he presented with severe failure to thrive, secondary to malabsorption, at three months of age. His weight at the time was 2.77 kg (in comparison to his birth weight of 2.255 kg) and his length was 50 cm, placing him significantly below the third percentile for both weight and height [17]. In addition, there was a history of chronic diarrhoea, with an average of eight bulky stools of semi-solid consistency being passed daily, along with chronic vomiting. Stool elastase levels were decreased at 107 μg/mL (normal: >200 μg/mL), consistent with mild to moderate pancreatic insufficiency. Furthermore, the patient was positive for serum immunoreactive trypsinogen (IRT); subsequent *CFTR* gene sequencing did not reveal any pathogenic variants, rendering a diagnosis of cystic fibrosis unlikely. Other laboratory investigations showed moderately increased AST and ALT enzyme levels. Further information regarding possible endocrine pancreatic dysfunction was unavailable.

Taken together, the bulky diarrhoea, positive IRT and low stool elastase levels pointed to a diagnosis of chronic pancreatitis with exocrine pancreatic insufficiency. The patient was therefore commenced on pancreatic enzyme replacement therapy (2000 units of pancrelipase (Creon) six hours before feeding) and supplementation of fat-soluble vitamins A, D, E and K. Subsequently, at age 3 years and 7 months, the patient represented with severe failure to thrive and feeding intolerance. His weight on admission was 5.75 kg, again significantly below the 3rd percentile, suggesting a lack of benefit from pancreatic enzyme replacement. He subsequently developed a severe infection and passed away two weeks later, with cause of death attributed to sepsis on background of severe failure to thrive. The patient never underwent CT imaging but had four abdominal X-rays performed between two and fourteen months of age, for identification of the position of enteric feeding tubes. These investigations were unremarkable with regards to the pancreas or other clinically significant findings.

### 3.3 Case 3

Patient 3 is a 17-year-old-patient with an established diagnosis of an *EPG5*-related disorder due to homozygosity for the recurrent Ashkenazi *EPG5* founder variant NC_000018.10:g.45954395 T > C;NM_020964.3:c.1007 A > G;NP_066015.2:p.Gln336Arg. He had global developmental delay and seizures. He also suffered from recurrent, predominantly sinopulmonary, infections suggestive of an underlying immunodeficiency, although this was not formally confirmed on specific laboratory investigations. He showed consistently elevated amylase levels suggestive of pancreatic involvement for which no alternative cause was identified. In particular, from the age of 12 years serum amylase levels were found to be consistently above 3 times the upper limit of the normal range for more than a year, with the lowest level 532 IU/L and the highest level 769 IU/L (normal range 30–110 IU/L). Isoenzyme testing confirmed the pancreatic origin of this elevated serum amylase: serum levels of the salivary isoenzyme were repeatedly measured at 0 IU/L (normal range 4–61 IU/L). Serum amylase levels had come down to 384 IU/L and normalised after 18 months. Interestingly, serum lipase measurements during this period were normal, or only slightly above the upper normal limit. In addition, the patient experienced multiple acute episodes suggestive of pancreatic involvement, including a presentation with severe abdominal pain at the age of 6 years prompting a review at the emergency department where an abdominal CT scan revealed mesenteric oedema but no other underlying cause. At the age of 10 years, he experienced significant but variable abdominal pain over a 6-month period; during these episodes, serum amylase levels trended towards the upper limit of normal, up to 109 IU/L.

## 4 Literature review

A literature review was done by searching the terms “autophagy”/”EPG5”/”Rab7”/other autophagy-linked genes identified in the search AND “pancreas”/”pancreatitis” in PubMed, for papers published between 01/01/1980 and 01/06/2024. Findings from the literature review are discussed below and a summary of autophagy-related genes known to play a role in pancreatitis pathophysiology is provided in Table 1.

**Table 1:** The role of autophagy-related genes implicated in pancreatitis.

Gene	Function in autophagy	Pancreatic phenotype with loss-of-function	Features of related pancreatitis	Model	References
*ATG5*	Autophagosome formation	Atrophic chronic pancreatitis and endocrine pancreatic insufficiency	Accumulation of defective mitochondria, increased ER stress, ROS generation.	Mouse *in vivo*	[48]
*ATG7*	Autophagosome formation	Acute pancreatitis progressing to chronic pancreatitis	Zymogen granule accumulation	Mouse *in vivo*	[26]
*IKKα*	Positive regulator of autophagy induction	Spontaneous pancreatitis with progression to chronic pancreatitis	Accumulation of misfolded protein aggregates, increased UPR and ER stress, accumulation of swollen, fragmented mitochondria	Mouse *in vivo*; human pancreas samples	[45]
*GNPTAB*	Key enzyme in M6P tag formation that targets lysosomal enzymes to lysosome	Spontaneous pancreatitis in mice, possible chronic pancreatitis in human patients	Failure of autophagosomal processing leads to accumulation of defective mitochondria, Impaired ATP synthesis	Mouse *in vivo*; autopsy findings in human patient	[50,68]
*TFEB*	Transcription factor upregulating lysosomal genes	Acute pancreatitis and increased susceptibility to alcoholic pancreatitis (overexpression protective)	Decreased lysosomal biogenesis with impaired clearance of zymogen granules	Mouse *in vivo*	[66]
*LAMP-2*	Autophagosome-lysosome fusion (*LAMP-1 is similar but deletion causes no phenotype where LAMP-2 is intact).*	Chronic pancreatitis without fibrosis (LAMP-2 necessary for stellate cell function)	Massive vacuolisation, shift from apoptosis to necrosis, chronic inflammation	Mouse *in vivo*	[20,36,37]
*RAB7*	Autophagosome-lysosome fusion specificity	Exacerbated severity of experimental acute pancreatitis	Increased autophagic vacuole formation and increased cathepsin B expression (activates trypsin).	Mouse *in vivo*	[32]
*VMP1*	Upregulates autophagic degradation of zymogen granules	Reduced protection from experimental pancreatitis	Loss of zymogen granule degradation; excessive trypsin activation	Mouse *in vivo*	[27]
*CCPG1*	Promotes ER-phagy in response to ER stress/UPR	Loss of pancreatic proteostasis with increased ER-stress; chronic pancreatitis-like features	Distended ER, increased UPR, inflammatory infiltrates and acinar cell death	Mouse *in vivo*	[67]

M6P = mannose-6-phosphate. ER = endoplasmic reticulum. UPR = unfolded protein response. ER-phagy = autophagy of the endoplasmic reticulum. Mitophagy = autophagy of mitochondria.

## 5 Discussion

Here we present three patients with a principal diagnosis of *EPG5*-related Vici syndrome, the paradigmatic disorder of defective autophagy, and additional pancreatic involvement, ranging from pauci-symptomatic serum amylase elevations to acute pancreatitis and chronic pancreatitis with pancreatic insufficiency. In all 3 patients, mutations in *CFTR* and other pancreatitis-associated genes were excluded, indicating that no other known genetic background was likely to be implicated in their pancreatic presentations. These findings have important implications for the health surveillance of patients with Vici syndrome, indicate a role of *EPG5* in pancreatic health, and may further serve in the elucidation of the pathophysiology of acute and chronic pancreatitis. Moreover, our literature search based on the criteria defined previously suggested multiple roles of autophagy in pancreatic health and disease as detailed below.

Vici syndrome is one of the most extensive human multisystem disorders reported to date. Whilst the focus so far has been mainly on neurodevelopmental and neurological aspects of this rare condition, virtually any organ or tissue may be affected, reflective of the ubiquitous expression and fundamental roles of the defective protein. Although there is the possibility that the presence of pancreatic involvement in our Vici syndrome patients may be coincidental, there are several lines of evidence to suggest that the association is indeed genuine: Pancreatitis has been previously reported in one other *EPG5*-mutated patient [18] (in a case series describing an attenuated form of Vici syndrome), strengthening our hypothesis of a potential causative link between EPG5 deficiency and pancreatic involvement. Moreover, the frequency of pancreatitis in our cohort of 211 patients identified through an international collaboration on *EPG5*-related disorders [5] (*n* = 3/211 or 1.4 %) is higher than what would be expected based on epidemiological data suggesting an annual acute pancreatitis incidence of 0.78 in 100,000 in UK children below 15 years [19]; this is even before taking into account that a large proportion of those included in our cohort died before the age of 2 years, and that some episodes of pancreatitis may have gone unnoticed in these profoundly delayed children. Finally, our findings are also in keeping with the suggestion that pancreatic involvement may be a feature in other autophagy-related disorders closely linked to Vici syndrome, for example in Danon disease, where the relevant mouse model shows features of chronic pancreatitis [20] although no humans with similar features have been reported to date. The hypothesis of a potentially more widespread pancreatic phenotype in autophagy-associated and related vesicular trafficking disorders is also supported by the observation of frequent pancreatic involvement in cystinosis [21], another condition with some phenotypical overlap with *EPG5*-related Vici syndrome and prominent autophagy involvement.

**Fig. 1:**
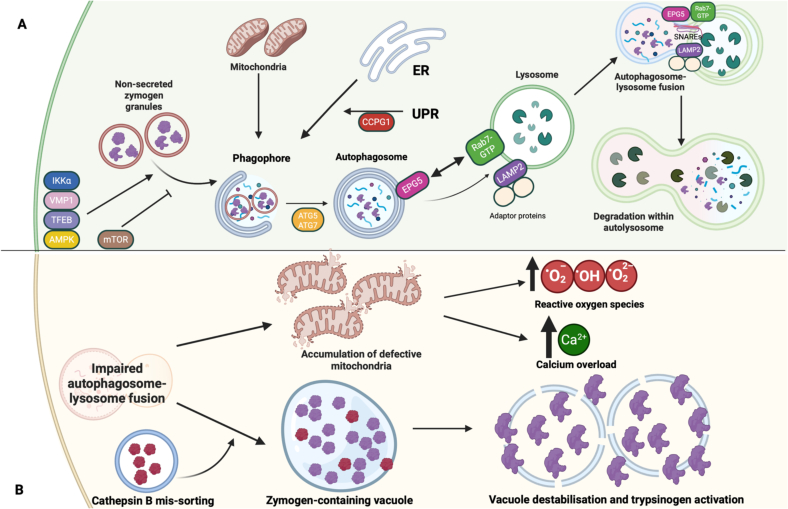
A. Summarises the role of autophagy in healthy pancreatic acinar cells. The interaction of EPG5 with Rab7-GTP is essential for correct targeting of autophagosomes to lysosomes and subsequent SNARE protein interaction. LAMP2 is a lysosomal membrane protein that recruits additional adaptor proteins essential for the process. The roles of other relevant proteins are summarized in Table 1 and detailed in the Discussion. ER = Endoplasmic reticulum. UPR = Unfolded protein response. B. provides a simplified summary of how defective autophagy can promote pathologic changes and pancreatitis, as further detailed in Fig. 2.

**Fig. 2:**
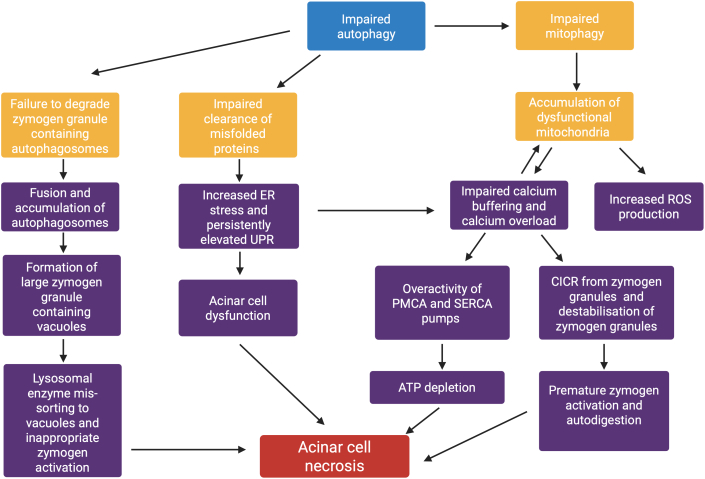
A flow chart summarising the role of defective autophagy in the pathophysiology of acute and chronic pancreatitis. ER = endoplasmic reticulum. UPR = unfolded protein response. ROS = reactive oxygen species. CICR = calcium-induced calcium release. PMCA = plasma membrane calcium ATPase. SERCA = sarco/endoplasmic reticulum calcium ATPase. ATP = adenosine triphosphate. Mitophagy = autophagy of mitochondria.

Autophagy is a highly structured and conserved intracellular pathway involving the delivery of defective intracellular cargo through specialized double-membraned structures, autophagosomes, to the lysosome for digestion and recycling. In addition to its general intracellular housekeeping roles, autophagy also fulfils more specialized roles in various tissues and organs, including the pancreas. These specifically pancreatic roles of autophagy identified through a topical literature search are detailed in the paragraph below, illustrated in Fig. 1 and summarized in Fig. 2.

*Abnormal vacuole formation and inappropriate trypsinogen activation in acinar cells* are two crucial events in the pathogenesis of pancreatitis and can be directly linked to abnormalities of autophagy and intracellular trafficking: In acinar cells, efficient autophagic flux results in the fast turnover of normally-sized or small autophagic vacuoles, whereas impaired autophagic results in the persistence of these vacuoles [22,23]. The vacuoles formed in acute pancreatitis, although much larger than those formed in response to fasting, share many features with autophagosomes, including the autophagosome marker Atg8 [24,25], suggesting they are derived from zymogen-containing autophagosomes that have failed to be degraded due to impaired autophagic flux [26], resulting in their accumulation. Starvation-induced increases in autophagy typically result in a significantly decreased intra-acinar zymogen-containing granules (ZGs) [25], suggesting that pancreatic autophagy induction may protect from inappropriate intra-acinar zymogen activation through the effective sequestration and degradation of ZGs [27]. If autophagic flux is blocked, however, these autophagic vacuoles may fuse and accumulate, potentially leading to inappropriate trypsinogen activation. This hypothesis is supported by increased levels of Rab7 in ZG-rich subcellular fractions, suggesting co-localisation of ZG-rich autophagosomes with lysosomes but ineffective lysosomal clearance in experimental models of disease. Importantly, these autophagosomal vacuoles are sites of trypsinogen activation [28], a key initiating event in acute pancreatitis. Zymogen granule accumulation due to impaired autophagic flux has also been shown to lead to the development of chronic pancreatitis [26,29]. Defective autophagic flux may cause an increase in cathepsin B (an activator of trypsinogen) activity relative to cathepsin L (a degrader of active trypsin), promoting intra-acinar trypsin activity and acinar autodigestion [23]; this effect may be further enhanced through mis-sorting of cathepsin B to ZG-containing autophagic vacuoles, the site of impaired autophagosome degradation where cathepsin B may catalyse the activation of trypsinogen to trypsin directly [30].

A more specific link between genetically impaired autophagy and abnormalities of pancreatic function is further supported by observations in relevant animal models: In mice deficient in Rab7, a protein acting in concert with EPG5 to ensure autophagosome-lysosome fusion specificity [31], expression of cathepsin B has also been demonstrated to be significantly elevated compared to wild type mice [32]. While a similar effect on pancreatic cells has not yet been specifically investigated in mouse models of EPG5 deficiency, further follow-up studies are needed in patients with *EPG5*-related Vici syndrome and corresponding animal models [33].

There is also more specific evidence implicating *impaired autophagosome-lysosome fusion* in the pathophysiology of pancreatitis [34]. For instance, the expression of lysosomal membrane protein *LAMP2*, essential for autophagosome-lysosome fusion, is required for acinar cell homeostasis in mice, with deficiency resulting in unstimulated digestive enzyme release, a predisposing factor for acute and chronic pancreatitis [20,35]. Consequently, *LAMP2*-deficient mice, a model of X-linked recessive Danon disease in humans, develop features strongly resembling chronic pancreatitis, including tissue disorganisation, macrophagic infiltration and stellate cell activation, and demonstrate substantially decreased intra-pancreatic levels of digestive enzymes, strongly suggestive of pancreatic insufficiency. These specific changes in the pancreas of *LAMP2*-deficient mice are also associated with the intra-acinar accumulation of autophagic vacuoles [36], as well as stalled autophagy in the exocrine pancreas and a shift in cell death patterns from non-inflammatory apoptosis to pro-inflammatory necrosis [37].

Defects in autophagy, an important source of ATP for many cell types [38], may also worsen the *ATP depletion* occurring in pancreatitis and thus further promote necrosis. Moreover, an increase in ADP:ATP ratios during acute pancreatitis may induce AMPK, an activator of autophagy, potentially leading to an increased accumulation in stalled autophagosomes [39].

Autophagy is also important in other aspects of acinar cell homeostasis such as recycling and clearance of misfolded proteins [40]. which appear frequently in acinar cells given their high protein synthesis rate. In autophagy-deficient cells, the consequently highly elevated unfolded protein response generates significant *endoplasmic reticulum (ER) stress* which can predispose cells to calcium overload [41], especially in the context of concurrent mitochondrial dysfunction. Elevated ER stress is an important mechanism of pancreatitis in humans, as shown by the accumulation of misfolded PRSS1 leading to hereditary chronic pancreatitis [42], and may lead to a reduction in acinar cell protein synthesis and ultimately cell death [43]. Autophagy is also an important mechanism for directly relieving ER stress through autodigestion of the expanded ER [44]. Mice defective in *IKKα*, a key signalling kinase in autophagy, have acinar cells significantly more prone to ER stress-induced cell death [45], creating a propensity for spontaneous pancreatitis, *via* a mechanism likely independent of abnormal trypsinogen activation [46]. Mice deficient in IKKα (and other key mediators of autophagy) also accumulate damaged and dysfunctional mitochondria [45], and the subsequent build-up of reactive oxygen species, as part of a vicious cycle, may therefore contribute to increased protein misfolding [47] and further ER stress. Another study in mice deficient in Atg5, a key protein in initiating autophagy, showed increased levels of reactive oxygen species in the pancreatic acini [48], contributing to cell necrosis and ultimately leading to the development of chronic atrophic pancreatitis. In all of these models of defective autophagy, the autophagy adaptor protein p62 was shown to accumulate intracellularly and demonstrably contribute to pathology, likely through activating pro-inflammatory signalling pathways [49].

Another mechanism that has been critically implicated in the pathogenesis of acute pancreatitis [50] is *mitochondrial dysfunction*, through a range of downstream effects [[51], [52], [53]]. Recurrent pancreatitis [54] and exocrine pancreatic insufficiency [55] are important (and occasionally presenting) features of human mitochondrial disease, highlighting the clinical relevance of mitochondrial dysfunction in pancreatitis. Defective autophagy plays a critical role in mitochondrial dysfunction observed in acute pancreatitis models [50], through its effects on mitophagy, an essential process for mitochondrial quality control [56] that has also recently been demonstrated to be affected in *EPG5*-related disorders [5]. When autophagy is defective, dysfunctional mitochondrial fragments accumulate in acinar cells with widespread consequences ultimately resulting in necrosis and apoptosis [57]. Mitochondrial dysfunction also predisposes acinar cells to the pathological calcium signalling that is characteristic of pancreatitis [58] due to reduced calcium buffering, a key protective function of mitochondria in acinar cells [59]. Inhibiting mitochondrial function in acinar cells converts the transient, oscillatory calcium waves triggered by secretory hormones into pathological, sustained calcium waves characteristic of pancreatitis [60]; an effect enhanced with primary *disturbances of calcium signalling* [61]. The calcium overload secondary to mitochondrial dysfunction has multiple downstream effects, including inflammation downstream of excessive calcineurin activation [62], ATP depletion by increased calcium ATPase pump activity [53], the destabilisation of zymogen granules and subsequent premature trypsinogen activation [63], resulting in a vicious cycle leading to further mitochondrial dysfunction [64].

Taken together, these observations suggest that patients with Vici syndrome may be at increased risk of developing pancreatic pathology, an aspect of the condition that has not been previously widely recognized. Our observations also provide clinical evidence from humans for a role of impaired autophagy in the development of both acute and chronic pancreatitis, a link so far only suggested in animal models of defective autophagy. Whilst autophagy is clearly not the only pathway implicated in pancreatic pathology, as outlined in more detail above it plays a crucial role in several aspects of pancreatic health and disease and is likely to have a detrimental effect if defective. Normal autophagosome-lysosome fusion in particular, the part of the autophagy pathway where EPG5 is critically involved, appears to play an essential role for the normal functioning of pancreatic processes, in line with the recent suggestion of a crucial role for EPG5 in the dysregulation of mannose-6-phosphate–dependent cholesterol homeostasis in pancreatic acinar cells [65].

In conclusion, our findings indicate that signs and symptoms of abdominal pathology (or even non-specific distress and discomfort) in children with Vici syndrome and other autophagy-related disorders should prompt appropriate laboratory and imaging investigations to exclude pancreatic involvement; considering that most of these patients are non-verbal, the threshold to request such investigations should be low and require a high degree of informed suspicion. As it is notoriously difficult to recognize novel disease associations in ultrarare diseases and some of those may have been underreported, our findings highlight the need for international collaborations focussing on wider clinical aspects of autophagy-related and other ultra-rare disorders.

## CRediT authorship contribution statement

**Dennis T. Famili:** Writing – review & editing, Writing – original draft, Formal analysis, Data curation, Conceptualization. **Gehad Elghazali:** Writing – review & editing, Data curation. **Emanuela Argili:** Writing – review & editing, Data curation. **Russell P. Saneto:** Writing – review & editing, Data curation. **Michael Harris:** Writing – review & editing, Data curation. **Oleg Gerasimenko:** Writing – review & editing, Writing – original draft, Formal analysis, Data curation, Conceptualization. **Julia Gerasimenko:** Writing – review & editing, Writing – original draft, Data curation, Conceptualization. **Manolis Fanto:** Writing – review & editing, Conceptualization. **Hormos Salimi Dafsari:** Writing – review & editing, Writing – original draft, Supervision, Formal analysis, Data curation, Conceptualization. **Heinz Jungbluth:** Writing – review & editing, Writing – original draft, Validation, Supervision, Project administration, Formal analysis, Data curation, Conceptualization.

## Declaration of competing interest

None.

## Data Availability

Data will be made available on reasonable request.
